# Comparison of tear protein levels in breast cancer patients and healthy controls using a *de novo* proteomic approach

**DOI:** 10.3892/or.2012.1849

**Published:** 2012-06-01

**Authors:** DANIEL BÖHM, KSENIA KELLER, JULIA PIETER, NILS BOEHM, DOMINIK WOLTERS, WULF SIGGELKOW, ANTJE LEBRECHT, MARCUS SCHMIDT, HEINZ KÖLBL, NORBERT PFEIFFER, FRANZ-HERMANN GRUS

**Affiliations:** 1 Department of Obstetrics and Gynecology, University Medical Center of the Johannes Gutenberg University Mainz, Langenbeckstrasse 1, D-55131 Mainz, Germany; 2 Experimental Ophthalmology, Department of Ophthalmology, University Medical Center of the Johannes Gutenberg University Mainz, Langenbeckstrasse 1, D-55131 Mainz, Germany

**Keywords:** breast cancer, biomarker, diagnosis, proteomics, tear fluids

## Abstract

Noninvasive biomarkers are urgently needed for early detection of breast cancer since the risk of recurrence, morbidity and mortality are closely related to disease stage at the time of primary surgery. In the past decade, many proteomics-based approaches were developed that utilize the protein profiling of human body fluids or identification of putative biomarkers to obtain more knowledge on the effects of cancer emergence and progression. Herein, we report on an analysis of proteins in the tear fluid from breast carcinoma patients and healthy women using a *de novo* proteomic approach and 25 mixed samples from each group. This study included 25 patients with primary invasive breast carcinoma and 25 age-matched healthy controls. We performed a MALDI-TOF-TOF-driven semi-quantitative comparison of tear protein levels in cancer (CA) and control (CTRL) using a *de novo* approach in pooled samples. Over 150 proteins in the tear fluid of CTRL and CA were identified. Using an in-house-developed algorithm we found more than 20 proteins distinctly upregulated or downregulated in the CTRL and CA groups. We identified several proteins that had modified expression in breast cancer patients. These proteins are involved in host immune system pathways (e.g., C1Q1 or S100A8) and different metabolic cascades (ALDH3A or TPI). Further validation of the results in an independent population combined with individual protein profiling of participants is needed to confirm the specificity of our findings and may lead to a better understanding of the pathological mechanism of breast cancer.

## Introduction

Breast cancer is still the leading cause of death in women worldwide (1). Although the detection rate of breast carcinoma has improved, many female patients die from metastatic relapse. Mammography is the best available method for detection of breast cancer after the age of 50; although, the detection rate of mammography is not as good in younger women due to their high density breast tissue (2). Early detection is beneficial in the fight against breast cancer. Currently, there are no clinical biomarkers available for early detection of breast cancer. Markers such as CA15.3 and CEA are useful, in combination with imaging and physical examination, for monitoring ongoing treatment in breast cancer patients with metastatic disease; although, they both lack the clinical specificity and sensitivity to be used routinely as a clinical diagnostic tool (3).

The development of high-throughput techniques in Proteomics expanded the search for new biomarkers and enabled the identification of proteins that may have a crucial role in emerging and progressing breast cancer. Proteome analysis of body fluids, such as sera, tear film, or urine, is a hot topic in Proteomics (4–6). Li *et al* found three differently regulated proteins in the sera of breast cancer patients and healthy subjects using surface-enhanced laser desorption/ionization time-of-flight based protein profiling in 2002 and Mathelin *et al* tried to validate these putative biomarkers, determining only two of them could be used for the discrimination of cancer patients (7,8) (reviewed in refs. 9,10). Some studies examined the nipple aspirate fluid of breast cancer patients and healthy patients (11). In 2005, Pawlik *et al* showed 17 distinctly regulated peptides; whereas, Li *et al* found different protein distribution patterns in the nipple aspirate fluid and ductal lavage with the use of SELDI-TOF mass spectrometry (12,13). Since then, many other protein profiling studies were published that used matrix-assisted laser desorption/ionization time-of-flight/time-of-flight mass spectrometry with differently regulated proteins (14–16). The advantage of the MALDI-TOF-TOF MS is the subsequent identification of the proteins of interest. In a previous study, we reported data from MALDI-TOF-TOF-based profiling of the sera that could distinguish breast cancer patients from age-matched healthy controls, and we could classify cancer patients with a high sensitivity of 89% (17).

Another proteomics-based approach for the exploration of cancer-derived differences is the highly-precise microarray platform. This approach can serve, instead of the common ELISA test, as a validation tool for the biomarkers identified from prior MALDI-TOF-TOF-based explorations of the proteome. Here, the antibodies are fixed on a highly-optimized surface. In this manner, several protein levels can be measured simultaneously due to the small required volume (nl) of the reagents. After fixation of the antibodies, the surfaces can be incubated with body fluids containing the appropriate proteins. This high-throughput technique is also very common for the profiling of carcinoma tissue or body fluids of diseased patients due to its miniaturized size, accuracy, and automated handling (18–20) (reviewed in ref. 21). Several comparative studies of breast cancer and healthy sera have been published. Our study group reported the regulation of several proteins were significantly different in the sera of breast cancer patients (22). The discovery of different protein patterns in diseased cohorts and control samples and subsequent identification of these biomarkers is a promising method of obtaining knowledge about the effects of several diseases (6,23,24). A well-developed and clinically proven biomarker signature could lead to early detection of cancer, which can have great benefits for patients.

Most proteomic studies of breast carcinoma published so far concentrate on profiling the tissue or body fluids near the emergence spot. Little is known about the proteome changes of distant body fluids. Some research groups examined the protein profiles of alternative body fluids such as urine or saliva and several differently regulated proteins were reported (reviewed in refs. 25,26). Previously, we showed different protein distributions in the tear fluid of breast cancer patients and healthy controls in a SELDI-TOF-based profiling study (17,27). Another comparative MALDI-TOF-TOF-driven analysis of healthy dog’s tear fluid and dogs diagnosed with cancer has been published (28). To our knowledge, no other comparative tear fluid proteomic studies for breast cancer have been reported. Tear fluid has unique properties as retrieval is minimally invasive and it does not contain as many highly-abundant proteins as serum.

Herein, we report a MALDI-TOF-TOF-driven semi-quantitative comparison of tear protein levels in cancer (CA) and control (CTRL) using a *de novo* approach in pooled samples. Using a signature of biomarkers significantly decreased or increased in groups of CA and CTRL could help to discriminate diseased women from the healthy population with high specificity and sensitivity and possibly lead to the establishment of a molecular diagnostic tool for breast cancer.

## Materials and methods

### Comparison of tear protein levels in pooled samples from CA and CTRL

This *de novo* study included 50 female subjects, 25 patients were diagnosed with primary invasive breast carcinoma and treated at the University Medical Center Mainz. At the time of diagnosis, none of the patients had developed distant metastases. Patients’ characteristics are summarized in Table I. The healthy control subjects were 25 age-matched women without any known malignancies who were treated at the University of Mainz medical center. All study members gave their informed consent for voluntary participation in this study. The protocols were approved by the institutional ethics committee in accordance with the ethical standard of Declaration of Helsinki (1964).

### Sample retrieval

Tear fluid was obtained from all participants using a Schirmer Strip. After the samples were drawn, the strips were frozen immediately at −80°C to prevent protein degradation. Tear proteins were prepared under strict and identical conditions for all patients. Prior to the experiments, the wet strip part was cut into small pieces and incubated with n-Dodecyl-β-D-maltoside overnight at 4°C with constant shaking. The next day, the eluates were briefly centrifuged and transferred into fresh tubes. All samples were stored prior to analysis at −20°C.

### Sample processing

For the comparison of protein levels in CTRL and CA, each of the 25 tear eluates were pooled together accordingly to the group and precipitated with three times the volume of acetone overnight at −80°C. The next day, tear proteins were centrifuged at 14000 × g and 4°C to prevent protein degradation. The supernatant was discarded and the proteins were resuspended in PBS. Protein concentrations were measured with the BCA Protein Assay kit (Thermo Scientific, Rockford IL, USA), according to the manufacturer’s protocol.

### 1D SDS-PAGE and sample purification

Pooled tear proteins (60 μg) from CTRL and CA were separated by molecular weight using 1D SDS-PAGE (gels, buffers, and equipment all purchased from Invitrogen, Darmstadt, Germany). After gel electrophoresis, the lanes were stained overnight and then rinsed with double-distilled water. In the next step, the lanes were subdivided into 32 bands and the proteins were digested with endopeptidase trypsin according to the modified digestion protocol from Shevchenko *et al*(29). For the purification and desalting of peptides, automated sample handling was preferred to reduce the fluctuations from measured proteins due to manual processing of the samples. The purification and the stepwise elution of the peptides with 10–50% acetonitrile were performed using C18 ZipTips (Millipore, Billerica, USA) on the Freedom EVO^®^, purification station (Tecan Group Ltd., Männedorf, Switzerland). The eluted proteins (3 μl) were directly spotted on the MALDI TOF/TOF polished steel target and coated with 3 μl crystallization matrix (20 mg cinnamic acid/50% acetonitrile/2% trifluoroacetic acid). The matrix included 0.5 μl of a Reserpine solution (1 mg/ml) dissolved in methanol for signal normalization. All samples were measured head-to-head to avoid protein degradation and measurement fluctuations in the MALDI-TOF/TOF mass spectrometer (UltraflexII, Bruker Daltonik GmbH, Bremen, Germany). The peak detection was performed with internal calibration mix (Peptide calibration standard II, Bruker Daltonik GmbH).

### Data processing

After MALDI-TOF/TOF measurements, the spectra were exported into Proteomics Pipeline Mainz (P^2^M) software, developed in-house, and normalized according to the Reserpine peaks. Proteins were identified using the MASCOT protein search tool (30). The Swissprot database was chosen for the identification of proteins (31). The following general parameters were used: carbamidomethyl as a global modification and oxidation (M) as a variable modification with an MS tolerance of 100 ppm and MS/MS tolerance of 0.8. Only one miscleaved site was allowed and the MudPIT scoring system was used. For further analysis of the protein regulation levels, the intensities of the peptides for each protein were summed and the ratio of the intensity between both groups was calculated for each protein. Significant differences in protein expression levels were defined as at least two times higher or lower expression than the other group. STRING and Cytoscape software were used for the analysis of protein-protein interactions (32,33).

## Results

In this study we conducted an explorative and comparative analysis of the tear proteome of breast carcinoma patients and age-matched healthy controls. We tried to minimize protein degradation and fluctuations of protein measurements to achieve a precise comparison of protein levels. One person performed the experimental steps for the preparation of tear samples for 1D SDS-PAGE until the transfer of digested fractions onto the sample plate for the robotic purification station. The peptide purification was performed automatically to avoid fluctuations due to the manual handling of samples. Likewise, the experimental steps from the precipitation of the tear eluates were also performed by the same person.

### Semiquantitative comparison of protein levels in CA and CTRL

**Figure 1:**
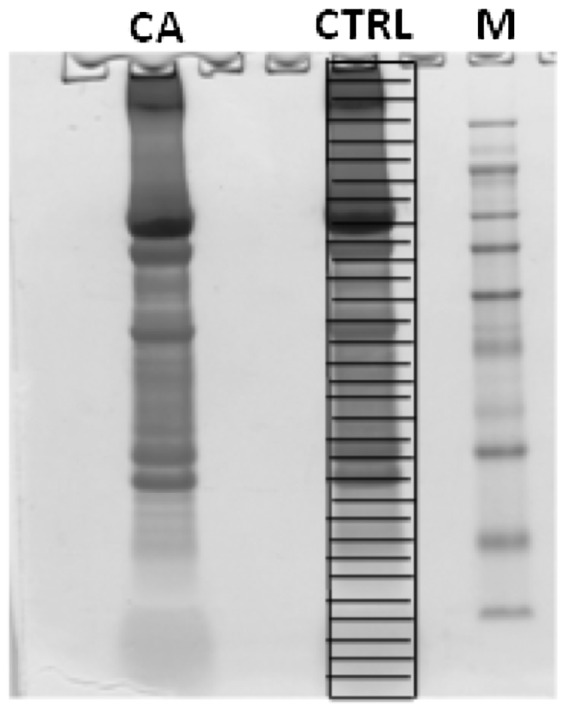
The stained tear samples from CTRL and CA. As an example, the composed grid is shown on the CTRL lane. The lane M shows the protein standard (SeeBlue^®^ Plus2 Pre-Stained Standard Invitrogen, Darmstadt, Germany).

**Figure 2:**
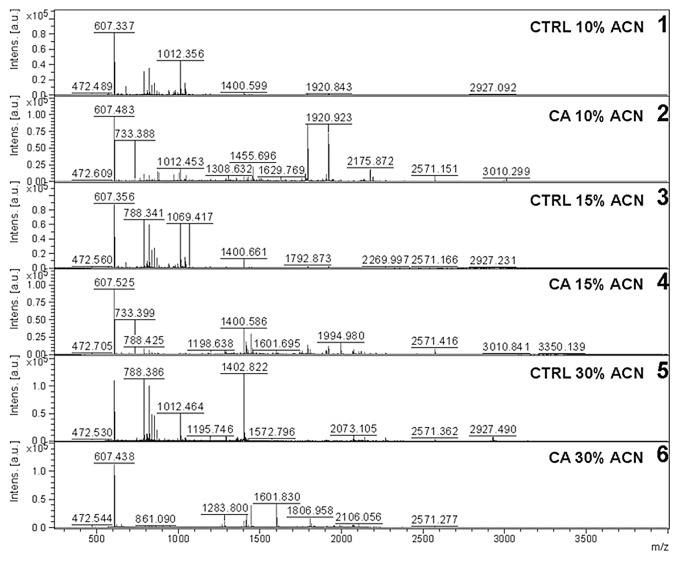
Mass spectra of the digested and purified peptides from the CTRL and CA groups. The molecular weight of the digested peptides is shown on the x-axis; while, the y-axis represents the intensity of the signals obtained. The spectra show the peptides derived from the first gel fraction in both groups. Spectra 1 and 2 show the peptides eluted with 10% acetonitrile (ACN). Spectra 3 and 4 demonstrate the peptides after elution with 15% ACN. Spectra 5 and 6 represent peptides from the elution step with 30% ACN. All spectra show the reproducible high intensity of signals. The mass spectra show appropriate differences in the peptide patterns according to the CTRL and CA pools.

After destaining, a grid made of 32 bands was put under the gel for a more accurate comparison of the proteins. Each of the 32 bands from CTRL and CA were cut out and digested with trypsin overnight. Fig. 1 shows the samples after 1D SDS-PAGE separation and staining with Coomassie dye (Colloidal Blue Staining kit, Invitrogen). After digestion and automated fractionation, the peptides were measured in a MALDI-TOF-TOF mass spectrometer. Representative fractions from both groups are shown in Fig. 2. All spectra were normalized using Protein Pipeline Mainz software, which was developed in-house, and the appropriate tear proteins were identified with the MASCOT search tool.

### Protein identification

**Figure 3:**
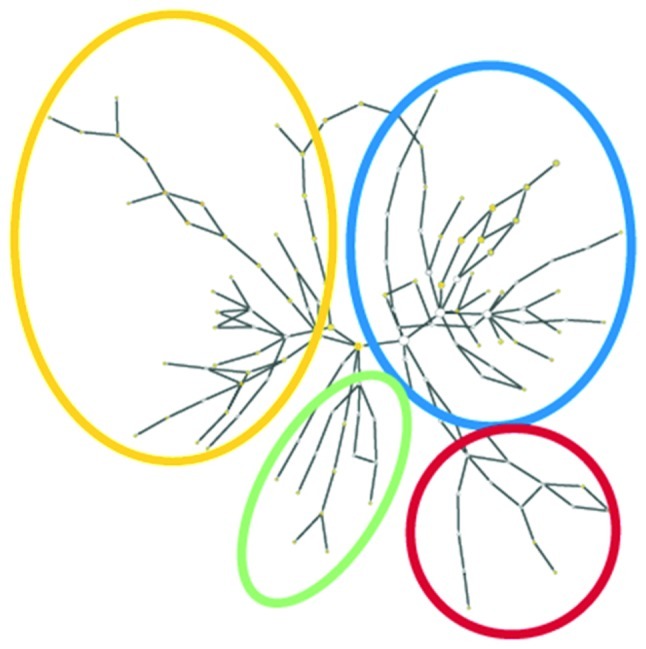
Network of identified proteins in the tear fluid of CA and CTRL. Using the software Cytoscape 2.7.0, we constructed a network of the merged proteins identified in both groups. In this overview, the proteins are clustered according to the molecular functions. The ontology file GO_Molecular_Function was used. Other criteria were: hypergeometric statistic test, accessing overrepresented categories, and significance level of 0.05. The circled areas summarize the identified proteins and their molecular functions as follows: Yellow circle: histone modification, transferase activity; blue circle: binding of DNA, lipids and proteins, transcription activation; red circle: transmembrane transport activity; and green circle: catalytic activity. The yellow color on the nodes indicates a higher number of the assigned proteins.

After extensive comparison of the spectra obtained using the annotated proteins in the SWISSPROT Homo sapiens database under the given conditions and MOWSE score, we were able to identify over 150 proteins in the CTRL and CA. The complete merged list of identified proteins is summarized in Table II. To obtain an overview on the relevance and role of the identified proteins, we clustered the proteins in accordance to their molecular functions using the software Cytoscape 2.7.0, as shown in Fig. 3. The Cytoscape software often shows several overlapping molecular functions and distributions into several biological processes; therefore, we created an overview of one mapping possibility for a large number of the identified tear proteins.

**Figure 4:**
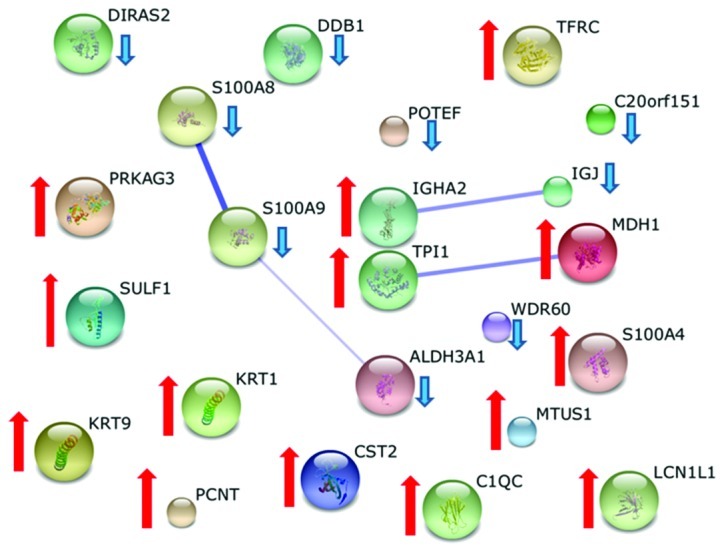
Using the software, STRING, we constructed an overview of the proteins that were at least 2-fold differently regulated in CTRL and CA. The appropriate gene names are abbreviated. The arrows show the increase (red arrow) or decrease (blue arrow) of the proteins in CA. Some of the known interactions of the proteins are shown with connection lines. The thickness of the lines shows how strong the interactions are.

Using the in-house-developed algorithm, we compared the protein levels in both groups. More than 20 proteins were distinctly upregulated or downregulated in the CTRL and CA groups and were involved in many biological processes such as metabolism (ALDH3A or TPI) or immune response (e.g., C1Q1 or S100A8). Table III shows a detailed list of the increased or decreased proteins in the tear fluid of breast cancer patients. Of note, the findings include inflammation proteins or complement factors for pathologic processes such as cancer that have already been described (34–36). Moreover, several proteins show at least four-fold higher (Extracellular sulfatase Sulf-1, Cystatin SA, cst2; 5-AMP-activated protein kinase subunit gamma-3, prkag3; Triosephosphate isomerase, tpi1; Microtubule-associated tumor suppressor 1, mtus1; Transferrin receptor protein 1, trfc; and Putative lipocalin 1-like protein 1, lcn1l1) or lower levels (DNA damage-binding protein 1, ddb1; Protein S100-A9, s100a9; and GTP-binding protein Di-Ras2, diras2) in CA. An overview of the proteins differently regulated in the CA group was constructed according to their regulation using the STRING tool and is shown in Fig. 4(32).

## Discussion

Data from high-throughput proteomic technologies, such as SELDI-TOF MS, MALDI-TOF-TOF MS, and microarray platforms, have recently increased. These techniques allow simultaneous protein profiling and subsequent identification of proteins and their subunits (5,37,38). A huge number of proteome studies have been published for proteome comparison of cancer patients and controls. Likewise, different proteomic studies reported significant differences in protein levels in the body fluids of breast cancer patients and healthy subjects (38,39). In our study, we concentrated on the tear proteome for several reasons. First, the sample retrieval is minimally invasive for the participants and tear fluid is easy to obtain with a simple Schirmer test. Second, the tear proteome contains no high-abundant proteins, such as albumin and immunoglobulins that are found in serum; therefore, it is not necessary to perform additional depletion steps that may cause distortion of potentially important proteins. In addition, we find it very intriguing to explore the tear proteome for potential biomarkers of breast cancer as it is an uncommon approach.

Some of the differently regulated proteins in our *de novo* pooled experiment have been reported to be altered in the tear fluid of patients with ophthalmic disease. Zhou *et al* reported S100A8 and S100A9 are increased in patients with dry eyes and Grus *et al* reported an increase in protein S100A8 (34,40). Both proteins belong to the family of S100 calcium-binding proteins, whose members seem to be involved in pro-inflammatory pathways as previously reported by Nacken *et al*(35). Some of the proteins may be of high interest, e.g., Mitochondrial tumor suppressor 1, MTUS1 and DNA damage binding protein, DDB1. MTUS1 regulates the cell cycle by acting as a tumor suppressor and DDB1 is involved in nucleotide excision repair. In addition, many of the differently regulated proteins are involved in metabolic processes, e.g., TPI or MDH1 in glycolysis and the citric acid cycle, which are both increased in the tear fluid of cancer patients. However, higher levels of autoantibodies against TPI1 have been reported in the sera of breast cancer patients (36). In our previous studies, we found several alterations in protein expression in the sera and tear fluid of breast cancer patients (22,41). Further analysis of the SELDI-TOF-based tear proteome profiling identified the protein S100A4 to be increased in the tears of breast cancer patients (data not shown). This result was confirmed in this study. The protein S100A4 was also previously found to be upregulated in patients with dry eye syndrome (40). Noteworthy, we observed several alterations in the level of proteins involved in immune response, such as complement factor C1Q1 or fragments of immunoglobulins (Table II). Also, several complement factors have been reported to be differentially regulated in the sera of cancer patients (42,43). Although, some of the results were controversial and may have resulted from different storage and handling conditions (44). Thus, members of the complement system may have additional roles. Markiewski *et al* reported tumor growth was promoted by C5a in their experiments with a cervical cancer mouse model (45,46).

To our knowledge, little is known about protein expression in the tear fluid of breast cancer patients. Only a very small number of tear proteome studies concerning proteome changes during breast cancer or cancer in general have been published. Further subsequent analyses and validation of our results in a tear protein study with an independent population and a higher number of participants will follow that also includes individual profiling. The findings from this study are intriguing as they may deepen the understanding of the impact of cancer and several cancer-driven pathways. Our study demonstrates that different biological processes are altered not only in prominent and broadly investigated body fluids such as serum and plasma, but also in discrete fluids such as tears that are located far away from the cancer site. As we already mentioned, several proteins have been reported to be modified in various types of body fluids, such as nipple aspirate fluid or urine. Our pilot study adds to these findings and shows again the complexity and multiple impacts of breast cancer while emerging and developing in the host, affecting biological processes and signal cascades. Moreover, we propose that a biomarker panel consisting of different proteins could accurately discriminate cancer patients from healthy controls. Therefore, it is important to examine the protein levels in an independent study population using individual protein profiling to validate our results. Further *de novo* approaches and validation of our results could lead to a better understanding of the pathological mechanism of breast cancer.

## Figures and Tables

**Table I:** Characteristics of breast cancer patients.

Characteristic	Breast cancer patients n=25 (%)	Healthy controls n=25
Mean age (distribution)	58 (39–85)	58 (39–85)
Tumor size		
pT1	16 (64)	
pT2	9 (36)	
Nodal status		
Negative	18 (72)	
Positive	7 (28)	
Grading		
Well differentiated (G1)	6 (24)	
Moderately differentiated (G2)	14 (56)	
Poor/undifferentiated (G3)	5 (20)	
Distant metastases		
M0	25 (100)	
M1	0 (0)	

**Table II:** Proteins identified from tear proteomes of CA and CTRL.

Protein	Description	Organism species (OS)	Gene name (GN)
TRFL_HUMAN	Lactotransferrin	Homo sapiens	LTF
LCN1_HUMAN	Lipocalin-1	Homo sapiens	LCN1
ALBU_HUMAN	Serum albumin	Homo sapiens	ALB
IGKC_HUMAN	Ig κ chain C region	Homo sapiens	IGKC
SG2A1_HUMAN	Mammaglobin-B	Homo sapiens	SCGB2A1
LYSC_HUMAN	Lysozyme C	Homo sapiens	LYZ
PIP_HUMAN	Prolactin-inducible protein	Homo sapiens	PIP
DMBT1_HUMAN	Deleted in malignant brain tumors 1 protein	Homo sapiens	DMBT1
IGHA1_HUMAN	Ig α-1 chain C region	Homo sapiens	IGHA1
IGHA2_HUMAN	Ig α-2 chain C region	Homo sapiens	IGHA2
GSTP1_HUMAN	Glutathione S-transferase P	Homo sapiens	GSTP1
ZA2G_HUMAN	Zinc-α-2-glycoprotein	Homo sapiens	AZGP1
ACTB_HUMAN	Actin, cytoplasmic 1	Homo sapiens	ACTB
CYTN_HUMAN	Cystatin-SN	Homo sapiens	CST1
LC1L1_HUMAN	Putative lipocalin 1-like protein 1	Homo sapiens	LCN1L1
PROL4_HUMAN	Proline-rich protein 4	Homo sapiens	PRR4
CYTS_HUMAN	Cystatin-S	Homo sapiens	CST4
ACTBL_HUMAN	β-actin-like protein 2	Homo sapiens	ACTBL2
POTEE_HUMAN	POTE ankyrin domain family member E	Homo sapiens	POTEE
POTEF_HUMAN	POTE ankyrin domain family member F	Homo sapiens	POTEF
ACTC_HUMAN	Actin, α cardiac muscle 1	Homo sapiens	ACTC1
LAC2_HUMAN	Ig λ-2 chain C regions	Homo sapiens	IGLC2
SG1D1_HUMAN	Secretoglobin family 1D member 1	Homo sapiens	SCGB1D1
S10A9_HUMAN	Protein S100-A9	Homo sapiens	S100A9
K1C9_HUMAN	Keratin, type I cytoskeletal 9	Homo sapiens	KRT9
TMC8_HUMAN	Transmembrane channel-like protein 8	Homo sapiens	TMC8
K2C1_HUMAN	Keratin, type II cytoskeletal 1	Homo sapiens	KRT1
LAC1_HUMAN	Ig λ-1 chain C regions	Homo sapiens	IGLC1
CYTT_HUMAN	Cystatin-SA	Homo sapiens	CST2
PIGR_HUMAN	Polymeric immunoglobulin receptor	Homo sapiens	PIGR
S10A8_HUMAN	Protein S100-A8	Homo sapiens	S100A8
APOA1_HUMAN	Apolipoprotein A-I	Homo sapiens	APOA1
PROL1_HUMAN	Proline-rich protein 1	Homo sapiens	PROL1
HSPB1_HUMAN	Heat shock protein β-1	Homo sapiens	HSPB1
LACRT_HUMAN	Extracellular glycoprotein lacritin	Homo sapiens	LACRT
ABCA3_HUMAN	ATP-binding cassette sub-family A member 3	Homo sapiens	ABCA3
IGJ_HUMAN	Immunoglobulin J chain	Homo sapiens	IGJ
ANXA2_HUMAN	Annexin A2	Homo sapiens	ANXA2
SSH2_HUMAN	Protein phosphatase Slingshot homolog 2	Homo sapiens	SSH2
KV301_HUMAN	Ig κ chain V-III region B6	Homo sapiens	
KV307_HUMAN	Ig κ chain V-III region GOL	Homo sapiens	
TPIS_HUMAN	Triosephosphate isomerase	Homo sapiens	TPI1
LEG3_HUMAN	Galectin-3	Homo sapiens	LGALS3
NGAL_HUMAN	Neutrophil gelatinase-associated lipocalin	Homo sapiens	LCN2
POP1_HUMAN	Ribonucleases P/MRP protein subunit POP1	Homo sapiens	POP1
ZC3H1_HUMAN	Zinc finger C3H1 domain-containing protein	Homo sapiens	ZFC3H1
CLIC1_HUMAN	Chloride intracellular channel protein 1	Homo sapiens	CLIC1
LIME1_HUMAN	Lck-interacting transmembrane adapter 1	Homo sapiens	LIME1
HV307_HUMAN	Ig heavy chain V-III region CAM	Homo sapiens	
GNL3_HUMAN	Guanine nucleotide-binding protein-like 3	Homo sapiens	GNL3
POTEI_HUMAN	POTE ankyrin domain family member I	Homo sapiens	POTEI
ENOA_HUMAN	α-enolase	Homo sapiens	ENO1
PRDX1_HUMAN	Peroxiredoxin-1	Homo sapiens	PRDX1
MECP2_HUMAN	Methyl-CpG-binding protein 2	Homo sapiens	MECP2
K2C78_HUMAN	Keratin, type II cytoskeletal 78	Homo sapiens	KRT78
ZG16B_HUMAN	Zymogen granule protein 16 homolog B	Homo sapiens	ZG16B
YM012_HUMAN	Uncharacterized protein DKFZp434B061	Homo sapiens	
YV021_HUMAN	Uncharacterized protein LOC284861	Homo sapiens	
ILEU_HUMAN	Leukocyte elastase inhibitor	Homo sapiens	SERPINB1
ANXA1_HUMAN	Annexin A1	Homo sapiens	ANXA1
POTEJ_HUMAN	POTE ankyrin domain family member J	Homo sapiens	POTEJ
PLSL_HUMAN	Plastin-2	Homo sapiens	LCP1
NCOA5_HUMAN	Nuclear receptor coactivator 5, protein existence (PE), 1; sequence version (SV), 2	Homo sapiens	NCOA5
B2MG_HUMAN	β-2-microglobulin	Homo sapiens	B2M
KLH34_HUMAN	Kelch-like protein 34	Homo sapiens	KLHL34
ANX13_HUMAN	Annexin A13	Homo sapiens	ANXA13
MDHC_HUMAN	Malate dehydrogenase, cytoplasmic	Homo sapiens	MDH1
AIFM2_HUMAN	Apoptosis-inducing factor 2	Homo sapiens	AIFM2
STAG3_HUMAN	Cohesin subunit SA-3	Homo sapiens	STAG3
SMCA4_HUMAN	Transcription activator BRG1	Homo sapiens	SMARCA4
DDB1_HUMAN	DNA damage-binding protein 1	Homo sapiens	DDB1
RM18_HUMAN	39S ribosomal protein L18, mitochondrial	Homo sapiens	MRPL18
KRIT1_HUMAN	Krev interaction trapped protein 1	Homo sapiens	KRIT1
PERT_HUMAN	Thyroid peroxidase	Homo sapiens	TPO
HPT_HUMAN	Haptoglobin	Homo sapiens	HP
F184A_HUMAN	Protein FAM184A	Homo sapiens	FAM184A
AAKG2_HUMAN	5′-AMP-activated protein kinase subunit γ-2	Homo sapiens	PRKAG2
AAKG3_HUMAN	5′-AMP-activated protein kinase subunit γ-3	Homo sapiens	PRKAG3
EIF2A_HUMAN	Eukaryotic translation initiation factor 2A	Homo sapiens	EIF2A
RGPA2_HUMAN	Ral GTPase-activating protein subunit α-2	Homo sapiens	RALGAPA2
TUT4_HUMAN	Terminal uridylyltransferase 4	Homo sapiens	ZCCHC11
ATP4A_HUMAN	Potassium-transporting ATPase α chain 1	Homo sapiens	ATP4A
YJ017_HUMAN	Putative uncharacterized protein LOC439951	Homo sapiens	
AINX_HUMAN	α-internexin	Homo sapiens	INA
TTBK2_HUMAN	Tau-tubulin kinase 2	Homo sapiens	TTBK2
SPTN2_HUMAN	Spectrin β chain, brain 2	Homo sapiens	SPTBN2
MDGA1_HUMAN	MAM domain-containing glycosylphosphatidylinositol anchor protein 1	Homo sapiens	MDGA1
FREM3_HUMAN	FRAS1-related extracellular matrix protein 3	Homo sapiens	FREM3
PDE4C_HUMAN	cAMP-specific 3′,5′-cyclic phosphodiesterase 4C	Homo sapiens	PDE4C
SULF1_HUMAN	Extracellular sulfatase Sulf-1	Homo sapiens	SULF1
LRC4C_HUMAN	Leucine-rich repeat-containing protein 4C	Homo sapiens	LRRC4C
S10A4_HUMAN	Protein S100-A4	Homo sapiens	S100A4
LRFN6_HUMAN	Leucine-rich repeat and fibronectin type-III domain-containing protein 6	Homo sapiens	ELFN2
IGHG3_HUMAN	Ig γ-3 chain C region	Homo sapiens	IGHG3
IGHG2_HUMAN	Ig γ-2 chain C region	Homo sapiens	IGHG2
ELOA1_HUMAN	Transcription elongation factor B polypeptide 3	Homo sapiens	TCEB3
DLG3_HUMAN	Disks large homolog 3	Homo sapiens	DLG3
PDZD7_HUMAN	PDZ domain-containing protein 7	Homo sapiens	PDZD7
HV315_HUMAN	Ig heavy chain V-III region WAS	Homo sapiens	
HV304_HUMAN	Ig heavy chain V-III region TIL	Homo sapiens	
WBS23_HUMAN	Williams-Beuren syndrome chromosomal region 23 protein	Homo sapiens	WBSCR23
PKHH3_HUMAN	Pleckstrin homology domain-containing family H member 3	Homo sapiens	PLEKHH3
DMXL2_HUMAN	DmX-like protein 2	Homo sapiens	DMXL2
CBR3_HUMAN	Carbonyl reductase [NADPH] 3	Homo sapiens	CBR3
CE164_HUMAN	Centrosomal protein of 164 kDa	Homo sapiens	CEP164
USPL1_HUMAN	Ubiquitin-specific peptidase-like protein 1	Homo sapiens	USPL1
TRFE_HUMAN	Serotransferrin	Homo sapiens	TF
MPPA_HUMAN	Mitochondrial-processing peptidase subunit α	Homo sapiens	PMPCA
CABP1_HUMAN	Calcium-binding protein 1	Homo sapiens	CABP1
TFR1_HUMAN	Transferrin receptor protein 1	Homo sapiens	TFRC
ZN446_HUMAN	Zinc finger protein 446	Homo sapiens	ZNF446
MTDC_HUMAN	Bifunctional methylenetetrahydrofolate dehydrogenase/cyclohydrolase, mitochondrial	Homo sapiens	MTHFD2
CT151_HUMAN	Uncharacterized protein C20orf151	Homo sapiens	C20orf151
LIPB2_HUMAN	Liprin-β-2	Homo sapiens	PPFIBP2
ZSWM5_HUMAN	Zinc finger SWIM domain-containing protein 5	Homo sapiens	ZSWIM5
WDR60_HUMAN	WD repeat-containing protein 60	Homo sapiens	WDR60
C1QC_HUMAN	Complement C1q subcomponent subunit C	Homo sapiens	C1QC
CNOT1_HUMAN	CCR4-NOT transcription complex subunit 1	Homo sapiens	CNOT1
CDK13_HUMAN	Cyclin-dependent kinase 13	Homo sapiens	CDK13
GLE1_HUMAN	Nucleoporin GLE1	Homo sapiens	GLE1
RFIP4_HUMAN	Rab11 family-interacting protein 4	Homo sapiens	RAB11FIP4
AL3A1_HUMAN	Aldehyde dehydrogenase, dimeric NADP-preferring	Homo sapiens	ALDH3A1
FRMD7_HUMAN	FERM domain-containing protein 7	Homo sapiens	FRMD7
SEM4C_HUMAN	Semaphorin-4C	Homo sapiens	SEMA4C
PRTG_HUMAN	Protogenin	Homo sapiens	PRTG
PTPRR_HUMAN	Receptor-type tyrosine-protein phosphatase R	Homo sapiens	PTPRR
HV305_HUMAN	Ig heavy chain V-III region BRO	Homo sapiens	
TGS1_HUMAN	Trimethylguanosine synthase	Homo sapiens	TGS1
LRRK2_HUMAN	Leucine-rich repeat serine/threonine-protein kinase 2	Homo sapiens	LRRK2
BMPR2_HUMAN	Bone morphogenetic protein receptor type-2	Homo sapiens	BMPR2
F178A_HUMAN	Protein FAM178A	Homo sapiens	FAM178A
MOV10_HUMAN	Putative helicase MOV-10	Homo sapiens	MOV10
K0556_HUMAN	Uncharacterized protein KIAA0556	Homo sapiens	KIAA0556
KAT2A_HUMAN	Histone acetyltransferase KAT2A	Homo sapiens	KAT2A
EAP1_HUMAN	Enhanced at puberty protein 1	Homo sapiens	EAP1
CA175_HUMAN	Uncharacterized protein C1orf175	Homo sapiens	C1orf175
ENOG_HUMAN	γ-enolase	Homo sapiens	ENO2
ENOB_HUMAN	β-enolase	Homo sapiens	ENO3
LOX5_HUMAN	Arachidonate 5-lipoxygenase	Homo sapiens	ALOX5
MTMR4_HUMAN	Myotubularin-related protein 4	Homo sapiens	MTMR4
YQ050_HUMAN	Putative uncharacterized protein FLJ45831	Homo sapiens	
TRI75_HUMAN	Tripartite motif-containing protein 75	Homo sapiens	TRIM75
LRIG3_HUMAN	Leucine-rich repeats and immunoglobulin-like domains protein 3	Homo sapiens	LRIG3
DSCL1_HUMAN	Down syndrome cell adhesion molecule-like protein 1	Homo sapiens	DSCAML1
CD20_HUMAN	B-lymphocyte antigen CD20	Homo sapiens	MS4A1
IGHG4_HUMAN	Ig γ-4 chain C region	Homo sapiens	IGHG4
MIDA_HUMAN	Protein midA homolog, mitochondrial	Homo sapiens	C2orf56
SI1L3_HUMAN	Signal-induced proliferation-associated 1-like protein 3	Homo sapiens	SIPA1L3
TLE2_HUMAN	Transducin-like enhancer protein 2	Homo sapiens	TLE2
KLH17_HUMAN	Kelch-like protein 17	Homo sapiens	KLHL17
CO7A1_HUMAN	Collagen α-1(VII) chain	Homo sapiens	COL7A1
MRGRD_HUMAN	Mas-related G-protein coupled receptor member D	Homo sapiens	MRGPRD
MCF2L_HUMAN	Guanine nucleotide exchange factor DBS	Homo sapiens	MCF2L
MTUS1_HUMAN	Microtubule-associated tumor suppressor 1	Homo sapiens	MTUS1

**Table III:** Proteins increased or decreased at least 2-fold in CA.

A, Increased proteins in CA with fold increase		

Protein ID	Fold decrease	Number of compared peptides
Extracellular sulfatase Sulf-1	44	1
Cystatin-SA	9	2
5-AMP-activated protein kinase subunit γ-3	6	1
Triosephosphate isomerase	5.5	5
Microtubule-associated tumor suppressor 1	4.7	13
Transferrin receptor protein 1	4.5	6
Keratin, type I cytoskeletal 9	4.4	17
Putative lipocalin 1-like protein 1	4.1	1
Malate dehydrogenase, cytoplasmic	4	5
Ig α-2 chain C region	3.2	2
Ig heavy chain V-III region BRO	3.2	6
Protein S100-A4	3.2	1
Keratin, type II cytoskeletal 1	3.1	36
Pericentrin	2.8	49
Ig heavy chain V-III region WEA	2.7	2
Complement C1q subcomponent subunit C	2.6	1

B, Decreased proteins in CA with fold increase		

Protein ID	Fold decrease	Number of compared peptides

Aldehyde dehydrogenase, dimeric NADP-preferring	2.1	6
Immunoglobulin J chain	2.4	14
Ig γ-3 chain C region	2.4	12
POTE ankyrin domain family member F	2.5	6
Protein S100-A8	2.5	18
Uncharacterized protein C20orf151	2.9	9
Ig γ-4 chain C region	3	1
WD repeat-containing protein 60	3	3
DNA damage-binding protein 1	3.3	3
Protein S100-A9	3.3	11
GTP-binding protein Di-Ras2	10	1
